# 急性早幼粒细胞白血病合并弥散性血管内凝血1例报告并文献复习

**DOI:** 10.3760/cma.j.cn121090-20241130-00502

**Published:** 2024-12

**Authors:** 文静 谷, 荣凤 付, 磊 张, 仁池 杨, 晓帆 刘

**Affiliations:** 1 中国医学科学院血液病医院（中国医学科学院血液学研究所），血液与健康全国重点实验室，国家血液系统疾病临床医学研究中心，细胞生态海河实验室，天津 300020 State Key Laboratory of Experimental Hematology, National Clinical Research Center for Blood Diseases, Haihe Laboratory of Cell Ecosystem, Institute of Hematology & Blood Diseases Hospital, Chinese Academy of Medical Sciences & Peking Union Medical College, Tianjin 300020, China; 2 天津医学健康研究院，天津 301600 Tianjin Institutes of Health Science, Tianjin 301600, China; 3 中国医学科学院北京协和医学院群医学及公共卫生学院，北京 100730 School of Population Medicine and Public Health, Chinese Academy of Medical Sciences and Peking Union Medical College, Beijing 100730, China

## Abstract

急性早幼粒细胞白血病（APL）起病凶险，进展迅速，诱导化疗期间易合并弥散性血管内凝血（DIC），是患者早期死亡的主要原因。本文报道1例APL患者在维甲酸联合亚砷酸诱导化疗期间发生DIC，经积极血制品输注及凝血因子替代治疗，凝血异常得到纠正，随后经多疗程化疗获得完全缓解。本文对该患者的起病过程及治疗经过进行了详细描述，探讨了APL合并DIC与其他类型疾病所致DIC在发病机制、临床表现及治疗策略中的不同之处，并进行文献复习。

## Abstract (en)

Acute promyelocytic leukemia（APL）is characterized by an aggressive onset and rapid progression. During induction chemotherapy, it is prone to being complicated by disseminated intravascular coagulation（DIC）, which is a leading cause of early death in patients. This study reports a case of APL complicated by DIC during induction therapy with all-trans retinoic acid（ATRA）combined with arsenic trioxide（ATO）. The patient's coagulopathy was corrected through active transfusion of blood products and replacement therapy with coagulation factors. Subsequently, complete remission was achieved after multiple courses of chemotherapy. This article provides a detailed description of the patient's disease onset and treatment process, exploring the differences in pathogenesis, clinical presentation, and treatment strategies between APL-associated DIC and DIC caused by other diseases, alongside a review of the relevant literature.

急性早幼粒细胞白血病（APL）是一种由PML-RARα融合基因驱动的特殊亚型急性髓系白血病，具有独特的生物学特性和临床表现。由于大量早幼粒细胞释放促纤溶和促凝物质，APL患者极易合并弥散性血管内凝血（DIC），为诱导化疗期间早期死亡的主要原因[1]。APL凝血异常的早期识别和规范治疗至关重要。现介绍1例APL合并DIC患者的诊疗经过并进行相关文献复习。

## 病例资料

患者男性，44岁，2019年3月上呼吸道感染后出现低热，伴乏力症状，无皮肤黏膜或全身其他部位出血表现，于当地医院化验血常规示WBC 2×10^9^/L，后自觉症状好转，未进一步诊治。2019年4月11日患者因新发皮肤大片瘀斑就诊于我院门诊，复查血常规：WBC 1.08×10^9^/L、HGB 148 g/L、PLT 93×10^9^/L；尿隐血1+；ALT 87.6 U/L，AST 57.7 U/L；纤维蛋白原（FIB）1.43 g/L，纤维蛋白原分解产物（FDP）36.6 mg/L，D-二聚体13.47 mg/L。骨髓象：骨髓增生极度活跃，粒系比例增高，异常早幼粒细胞占有核细胞78.5％。外周血涂片异常早幼粒细胞1％。患者骨髓形态符合APL，4月12日开始予维甲酸20 mg每日2次诱导治疗，新鲜冰冻血浆、纤维蛋白原输注纠正凝血异常，4月15日收住院进一步治疗。患者骨髓检查陆续回报，骨髓活检：增生极度活跃，原始细胞弥漫增多（80％），网状纤维染色MF-1级。骨髓细胞实时定量PCR检测PML-RARα融合基因定量为88.54％，荧光原位杂交（FISH）检测PML-RARα融合基因阳性。患者明确诊断APL（低危组）。4月15日开始在维甲酸基础上加用亚砷酸10 mg/d双诱导治疗。4月15日患者WBC 1.1×10^9^/L，4月16日WBC 2.23×10^9^/L，加用羟基脲1 g每日3次，同时水化碱化、保护脏器功能支持治疗。血常规示白细胞呈升高趋势（3.09×10^9^/L→10.64×10^9^/L），于4月20日加用伊达比星10 mg+地塞米松10 mg/d。4月20日夜间患者突发胸闷、憋气、腹痛腹胀伴发热，实验室检查：WBC 31.43×10^9^/L、HGB 110 g/L、PLT 49×10^9^/L；ALT 126.2 U/L，AST 1114.7 U/L，肌酐221.7 µmol/L，尿酸855.5 µmol/L；肌酸激酶同工酶MB 13.6 µg/L，肌红蛋白500 µg/L；FIB 0.79 g/L，活化部分凝血活酶时间（APTT）30.6 s，凝血酶原时间（PT）28 s，FDP 557.2 µg/ml，D-二聚体>80 mg/L；降钙素原127.31 µg/L。胸部CT：双肺上叶炎症，双侧胸腔积液伴双肺膨胀不全。腹部CT：腹部肠管改变，不除外肠梗阻。停用维甲酸、羟基脲，予胃肠减压、抑酸解痉、抗感染，输注单采血小板、新鲜冰冻血浆、冷沉淀、纤维蛋白原、重组人凝血因子Ⅶa等纠正凝血异常。患者皮肤黏膜出血范围扩大，新发肌肉血肿，消化道及泌尿系出血。考虑患者病情进展，合并急性DIC、分化综合征（DS）、肿瘤溶解综合征、感染及多脏器衰竭，生命体征无法维持，于4月22日转入重症医学中心治疗，给予积极抗感染、止血、补充凝血因子及血液净化治疗。住院期间患者共输注单采血小板18个治疗量、纤维蛋白原23 g、新鲜冰冻血浆4800 ml、冷沉淀12 IU、悬浮红细胞26 IU。经治疗后患者出血及感染症状好转，生命体征趋于平稳，脏器功能恢复。5月8日患者重新采用维甲酸联合亚砷酸诱导治疗。6月5日复查骨穿，达到形态学及分子学完全缓解（CR），患者随后完成2疗程IA方案巩固治疗及后续维持治疗。随访至今，患者病情稳定，每年返院复查疗效评估均为CR。

## 讨论及文献复习

APL是一种特殊类型的急性髓系白血病，以t（15; 17）（q22; q12）染色体易位形成PML-RARα融合基因为特点[1]。多项临床研究报道APL诱导治疗期间的早期死亡（确诊后30 d内死亡）率为3％～10％，其中最常见的早期死亡原因为合并严重DIC而导致出血，可占全部死亡病例的41％～92％[2]–[6]。该患者依据中国DIC诊断积分系统（CDSS）[7]和国际血栓与止血学会（ISTH）的DIC诊断评分系统[8]，均达到DIC诊断标准。该患者诱导治疗期间病情变化迅速，十分凶险。因此，对APL合并DIC的深入研究有助于早期识别风险因素并进行及时有效干预。

DIC根据发病机制可分为4种不同类型。“出血型（高纤溶型）DIC”以纤溶亢进占主导地位，出血是其主要症状，多见于白血病、产科疾病、创伤、病毒性出血热及蛇毒所致DIC。“器官衰竭型（高凝低纤溶型）DIC”以高凝占主导地位，器官衰竭是其主要症状，常见于脓毒症、新型冠状病毒感染（COVID-19）、中暑所致DIC。当高凝和纤溶活性均很强时，若输血量不足，患者易发生大出血而死亡，该类型为“大出血型（消耗型）DIC”，常见于大手术后失血过多或危重产科疾病患者。当凝血和纤溶活性均很弱时，几乎没有临床症状，仅表现为凝血指标异常，该类型为“无症状型（临床前）DIC”[9]–[10]。各类“出血型”及“器官衰竭型DIC”的分子病理学机制见表1。该患者主要临床表现为皮肤/黏膜、肌肉及多脏器出血。实验室检查提示FIB减低，FDP及D-二聚体水平增高，PT延长，而抗凝血酶水平正常，属于典型的“出血型DIC”。

**表1:** 不同疾病所致DIC的病理学机制

DIC诱因	病理机制
出血型DIC	
APL	APL细胞高表达tPA、uPA和膜联蛋白Ⅱ，膜联蛋白Ⅱ对纤溶酶原及其激活剂均有高亲和力，促进纤溶酶原活化，增加纤维蛋白溶解
创伤	创伤出血所致血小板和凝血因子大量消耗；组织缺氧、低灌注引起低温和代谢性酸中毒，抑制凝血酶的活性，增加出血风险
大部分产科疾病	围产期出血或羊水栓塞导致血小板和凝血因子大量消耗；纤溶酶活性增强，纤维蛋白溶解产物抑制正常凝血
病毒性出血热	内皮损伤所致毛细血管通透性增加；炎症导致纤溶系统激活
蛇毒	毒液引起血小板和凝血因子大量消耗；毒液所致纤溶系统异常激活，纤维蛋白溶解亢进
器官衰竭型DIC	
脓毒症	凝血反应激活：单核细胞、血小板和内皮细胞受病原刺激表达促凝因子；抗血栓机制破坏：生理性抗凝系统受损；血管内皮损伤促进血栓形成；纤维蛋白溶解受抑制：PAI-1水平升高抑制纤溶系统
COVID-19	炎症风暴诱发内皮损伤；病毒的直接损伤作用：COVID-19与细胞表面的血管紧张素转换酶2结合，改变血小板功能和血管内皮的抗栓特性，促进血栓形成
中暑	高温引起炎症激活和内皮损伤

**注** DIC：弥散性血管内凝血；APL：急性早幼粒细胞白血病；tPA：组织型纤溶酶原激活物；uPA：尿激酶纤溶酶原激活物；PAI-1：纤溶酶原激活物抑制物1；COVID-19：新型冠状病毒感染

APL的凝血异常涉及多种复杂的病理生理机制，以原发性纤溶亢进为主，这也是与其他疾病合并DIC的不同之处[11]–[12]。APL细胞高表达组织型纤溶酶原激活物（tPA）、尿激酶纤溶酶原激活物（uPA）及其受体，而纤溶酶原激活物抑制物1和2（PAI-1和PAI-2）的表达减少。tPA和uPA均可激活纤溶酶原，导致纤维蛋白（原）溶解及凝血因子分解，引起出血。APL合并DIC的发生高峰期为诱导治疗后第一周内[13]，而本例患者在开始维甲酸诱导治疗第9天，降细胞治疗第5天发生DIC，较为少见。推测APL患者中迟发性DIC的可能机制为：①晚期凝血-抗凝失衡。某些患者的APL细胞经诱导治疗后分化较慢，治疗后期仍持续释放促凝物质，继续激活凝血级联。同时，治疗后期纤溶系统过度激活也进一步加剧了凝血-抗凝失衡[11]。②DS所致炎症风暴：DS多发生于APL患者诱导治疗开始后的7～14 d[14]，APL细胞分化为成熟粒细胞伴随大量炎性细胞因子的释放，炎症反应导致内皮损伤和凝血异常，诱发DIC[15]。③感染介导DIC：APL患者诱导分化及降细胞治疗后期患者免疫功能受抑，多合并各种病原微生物的机会性感染。感染相关炎症反应会激活凝血及纤溶系统，诱发DIC[16]。④肿瘤溶解综合征所致DIC：诱导分化导致持续APL细胞溶解破坏，释放大量胞内成分，酸中毒、组织缺氧及电解质代谢紊乱抑制血小板功能，诱发内皮损伤、凝血激活，导致DIC[17]–[18]。

初诊时高外周血原始细胞计数被多项研究公认为出血的主要预测因素[19]–[21]。其他与出血有不同程度关联的因素包括ECOG体能状态[21]、年龄[13]、血小板计数[22]、凝血指标（PT、APTT和D-二聚体水平）[13],[22]、乳酸脱氢酶[22]及血肌酐水平[13]。对APL出血相关预测因素的深入探索有助于提前发现高风险人群，在诱导治疗期间加强凝血因子替代治疗及支持治疗，将出血相关死亡风险降至最低。

临床上一旦疑诊APL，应立即启动全反式维甲酸治疗以降低致命性出血事件的发生风险[1]。对于已发生的DIC，参照欧洲白血病网（ELN）的建议，治疗包括替代治疗及药物治疗。替代治疗包括大量输注新鲜冰冻血浆、纤维蛋白原、冷沉淀及血小板，以维持纤维蛋白原浓度高于1.0～1.5 g/L，血小板计数高于30～50×10^9^/L以及国际标准化比值低于1.5。诱导治疗期间应至少每日监测血小板计数、PT、APTT、TT、FIB及FDP水平等，直至DIC临床症状消失且实验室指标正常[23]。药物治疗包括抗凝及抗纤溶。常见抗凝药物为普通肝素和低分子量肝素，其与抗凝血酶Ⅲ结合形成复合物，增强了对凝血因子（凝血因子Ⅹa为主）的抑制作用[24]。其中低分子量肝素抗凝作用弱，抗栓作用强，治疗相对安全，可明显减低APL合并DIC的早期死亡率[25]。因此建议在无活动性出血且充分补充凝血因子和血小板替代治疗的基础上应用低分子量肝素。因本例患者以出血为主，实验室检查指标符合典型的纤溶亢进表现，因此未选择抗凝治疗。常用的抗纤溶药物包括氨基己酸和氨甲环酸，可与纤溶酶原竞争性结合纤维蛋白原，与膜联蛋白α2和t-PA竞争性结合赖氨酸残基，起到抗纤溶作用，推荐用于纤溶亢进伴严重出血的患者[26]。另有学者认为抗纤溶治疗阻断了DIC的代偿机制，不利于组织器官灌注的恢复[27]，且回顾性分析表明抗纤溶治疗并未明显降低APL早期出血死亡率[28]。因此，本例患者未常规应用抗纤溶药物。

凝血酶原复合物和重组人凝血因子Ⅶa在某些报道中可用于APL伴危及生命出血的治疗[29]–[30]。重组可溶性血栓调节蛋白具有抗纤溶、抗炎及抗内皮损伤的作用，已在多个APL合并DIC研究队列中证实了有效性和安全性。因缺乏前瞻性研究数据，暂不推荐在临床试验之外使用该药物[31]–[32]。

APL合并DIC不同于其他疾病所致DIC，以原发性纤溶亢进为主要表现，患者可发生致命性大出血，为诱导治疗期间早期死亡的主要原因。初诊时高外周血原始细胞计数为公认的出血相关风险因素，有助于识别出血高风险人群并进行早期干预。APL合并DIC首选原发病的治疗。支持治疗包括输注血小板以及持续补充冷沉淀、新鲜冰冻血浆、纤维蛋白原等，以尽快使凝血功能恢复正常。若有危及生命的出血，可使用重组人凝血因子Ⅶa。抗凝及抗纤溶治疗对降低血栓出血的风险及获益需依据患者具体情况而定。
